# Regional Heritability Mapping to identify loci underlying genetic variation of complex traits

**DOI:** 10.1186/1753-6561-8-S5-S3

**Published:** 2014-10-07

**Authors:** Valentina Riggio, Ricardo Pong-Wong

**Affiliations:** 1 The Roslin Institute and the R(D)SVS, The University of Edinburgh, Easter Bush, Midlothian, EH25 9RG, Scotland, UK

## Abstract

**Background:**

Genome-wide association studies can have limited power to identify QTL, partly due to the stringent correction for multiple testing and low linkage-disequilibrium between SNPs and QTL. Regional Heritability Mapping (RHM) has been advanced as an alternative approach to capture underlying genetic effects. In this study, RHM was used to identify loci underlying variation in the 16^th ^QTLMAS workshop simulated traits.

**Methods:**

The method was implemented by fitting a mixed model where a genomic region and the overall genetic background were added as random effects. Heritabilities for the genetic regional effects were estimated, and the presence of a QTL in the region was tested using a likelihood ratio test (LRT). Several region sizes were considered (100, 50 and 20 adjacent SNPs). Bonferroni correction was used to calculate the LRT thresholds for genome-wide (p < 0.05) and suggestive (i.e., one false positive per genome scan) significance.

**Results:**

Genomic heritabilities (0.31, 0.32 and 0.48, respectively) and genetic correlations (0.80, -0.42 and 0.19, between trait-pairs 1&2, 1&3 and 2&3) were similar to the simulated ones. RHM identified 7 QTL (4 at genome-wide and 3 at suggestive level) for Trait1; 4 (2 genome-wide and 2 suggestive) for Trait2; and 7 (6 genome-wide and 1 suggestive) for Trait3. Only one of the identified suggestive QTL was a false-positive. The position of these QTL tended to coincide with the position where the largest QTL (or several of them) were simulated. Several signals were detected for the simulated QTL with smaller effect. A combined analysis including all significant regions showed that they explain more than half of the total genetic variance of the traits. However, this might be overestimated, due to Beavis effect. All QTL affecting traits 1&2 and 2&3 had positive correlations, following the trend of the overall correlation of both trait-pairs. All but one QTL affecting traits 1&3 were negatively correlated, in agreement with the simulated situation. Moreover, RHM identified extra loci that were not found by association and linkage analysis, highlighting the improved power of this approach.

**Conclusions:**

RHM identified the largest QTL among the simulated ones, with some signals for the ones with small effect. Moreover, RHM performed better than association and linkage analysis, in terms of both power and resolution.

## Background

Genome-wide association studies (GWAS) have generally failed to explain most of the known genetic variation influencing complex diseases [1]. This is partly due to the stringent correction for multiple testing and low linkage-disequilibrium (LD) between SNPs and QTL. Attempts to increase the power of GWAS have focused on increasing either the number of markers or the number of observations per trait. An alternative approach exploiting dense SNP chip data, known as Regional Heritability Mapping (RHM) [2], has been advanced as a better approach to capture more of the underlying genetic effects. This method provides heritability estimates attributable to small genomic regions, and it has the power to detect regions containing multiple alleles that individually contribute too little variance to be detected by GWAS. The aim of this study was to identify QTL affecting the three traits simulated in the 16^th ^QTL-MAS workshop dataset and recover their possible pleiotropic actions, using RHM.

## Methods

### 1. Dataset

The dataset, provided by the 16^th ^QTLMAS workshop organisers, consisted of 3,000 individuals, all females, from three generations (G1-G3); all were genotyped for about 10,000 SNPs on five chromosomes of equal length (99.95 Mb each). The phenotypes (Trait1, Trait2, and Trait3) resembled three milk production traits, given as individual yield deviations, and generated in order to mimic two yields and the corresponding content.

### 2. QTL mapping analysis

The implementation of RHM is described in [2]. RHM is related to interval mapping methodology, using variance component approach [3]. Basically, RHM is a mixed model where the effect of a genomic region (attributable to the QTL within the region in question) plus the overall genetic background were added as random, with covariance structure proportional to the genetic relationship matrix calculated using genotype information. The relationship matrix modelling the overall genetic background was estimated using all SNPs, whereas the one for the region was estimated using the SNPs falling within that region. Heritabilities for the genetic regional effects were estimated [4], and the presence of a QTL in the region was tested using a likelihood ratio test (LRT). Several region sizes were considered (i.e. 100, 50 and 20 adjacent SNPs), and the regions shifted every 10 SNPs. After Bonferroni correction, the LRT thresholds for genome-wide (p < 0.05) and suggestive (i.e., one false positive per genome scan) significance levels were 10.83 and 6.64 (corresponding to -log10(p) of 3.30 and 2.00), 12.12 and 7.88 (-log10(p) of 3.60 and 2.30), and 13.83 and 9.55 (-log10(p) of 4.00 and 2.70) for the three region sizes, respectively.

RHM results were compared with association and linkage analysis results, in order to assess its potential use as a tool for QTL mapping. The linkage analysis was implemented in GridQTL [5], studying the segregation of the paternal allele; the association analysis, using the GRAMMAR approach [6], which comprises two steps: first, phenotypes were adjusted for the polygenic effects and second, residuals were fitted against each SNP using additive model as implemented in GenABEL [7].

When a QTL was found significant for more than one trait, correlations between regional EBVs were estimated to evaluate possible pleiotropic effects among traits.

## Results and discussion

Heritabilities obtained using the genomic relationship matrix were 0.31, 0.32 and 0.48 for Trait1, 2 and 3, respectively and similar to those estimated with a pedigree-based relationship matrix (0.38, 0.39 and 0.49, respectively) and those simulated (0.36, 0.35 and 0.52, respectively). Genetic correlations estimated with the genomic relationship matrix were 0.80 (Trait 1&2), -0.43 (Trait 1&3) and 0.19 (Trait 2&3), and similar to those estimated with a pedigree-based relationship matrix (0.83, -0.42 and 0.14, respectively), and those simulated (0.80, -0.43 and 0.17, respectively).

**Figure 1:**
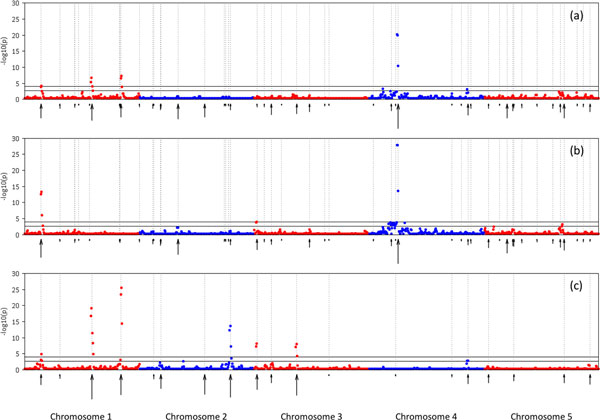
**RHM results for Trait1 (a), Trait2 (b) and Trait3 (c) using 20 SNP region size**. Genome-wide p < 0.05 and suggestive thresholds are shown (solid lines). Simulated QTL are also shown, with arrows whose size is proportional to the effect of the QTL.

The results from RHM were in general consistent across the three region sizes tested, hence, we will concentrate on the results with 20 SNPs per region. Figure 1 shows the Manhattan plot for the analyses of Trait1, 2 and 3 (a, b, and c, respectively). RHM identified 7 QTL (4 at genome-wide level and 3 at suggestive level) for Trait1; 4 (2 genome-wide and 2 suggestive) for Trait2; and 7 (6 genome-wide and 1 suggestive) for Trait3. Only one of the identified QTL, significant at the suggestive level, was a false-positive. The position of these QTL tended to coincide with the position where the largest QTL (or several of them) were simulated. RHM did not identify regions harbouring QTL with small effect, which is attributable to the size of the data. Nevertheless, several signals were detected, although they did not reach significance.

When comparing RHM results with association and linkage analysis results, all three methods were successful in identifying the larger QTL and with some extend the other ones (Additional Files 1 to 3). However, for the QTL with smaller effect RHM performed better than both association and linkage analysis, i.e. more power than association and more resolution than linkage.

Table 1 shows the heritability (h^2^_reg_) for all significant regions and the proportion of genetic variance explained when all significant QTL were simultaneously fitted into the model together with a genomic effect (to capture genetic variance not explained by the fitted QTL). The sum of all genetic variances from the joint analysis showed some discrepancies from the one observed in the analysis including one single genomic effect (especially with Trait3). Our results show that these regions explain more than half of the total genetic variance of the traits in question, suggesting that the undetected QTL explain a small proportion of genetic variance. However, the estimated variance might be inflated, due to Beavis effect [8], arising from small sample size.

**Table 1:** Regional heritability (h^2^_reg_) for regions significant both at the genomic level (p < 0.05) and at the suggestive level and proportion of genetic variance explained for the three traits with 20 SNP region size.

			Heritability (h^2^_reg_)		
**Chromosome**	**Region**	**Position (Kb)**	**Trait1**	**Trait2**	**Trait3**

1	30	14500-15450	0.02	0.03	0.03
	117	58000-58950	0.03		0.05
	169	84000-84950	0.02		0.05

2	159	79000-79950			0.01

3	5	2000-2950		0.03	0.03
	74	36500-37450			0.05

4	24	11500-12450	0.01^f.p.^		
	49	24000-24950	0.07	0.08	
	171	85000-85950	0.01		0.01

5	131	65000-65950	0.01	0.01	

**Sum of h^2^_reg_**			0.17	0.15	0.23
**Remaining heritability**			0.14	0.14	0.16
**Proportion of genetic variance explained by mapped QTL**			55%	52%	59%

^f.p. ^False-positive

Examination of the regional EBVs showed that some QTL have pleiotropic effects among traits. Genetic correlations (i.e., correlations between regional EBVs) between the regions in common across the three traits are in Table 2. All QTL affecting traits 1&2 and 2&3 had positive correlations, following the trend of the overall correlation of both trait-pairs. All but one QTL affecting traits 1&3 were negatively correlated. Our results were however in agreement with the simulated ones.

**Table 2:** Genetic correlations for regions which were found to affect more than one trait and overall genetic correlations.

			Genetic correlations		
			
Chromosome	Region	Position (Kb)	Trait 1&2	Trait 1&3	Trait 2&3
1	30	14500-15450	0.88	0.29	0.70
	117	58000-58950		-0.87	
	169	84000-84950		-0.89	
3	5	2000-2950			0.63
4	49	24000-24950	0.97		
	171	85000-85950		-0.85	
5	131	65000-65950	0.93		

**Overall correlation**			0.80	-0.43	0.19

## Conclusions

RHM identified the largest QTL among the simulated ones. Moreover, for the ones with smaller effect, several signals were detected, although they did not reach significance. In general RHM identified extra loci that were not found by association and linkage analysis, highlighting the improved power of this approach.

## Competing interests

The authors declare that they have no competing interests.

## Authors' contributions

VR carried out the analyses and drafted the manuscript; RPW implemented the method; both authors contributed to the design of the study and the final version of the manuscript.

## Supplementary Material

Additional file 1 **Comparison among RHM, association and linkage analysis results for Trait1**. Click here for file

Additional file 2 **Comparison among RHM, association and linkage analysis results for Trait2**. Click here for file

Additional file 3 **Comparison among RHM, association and linkage analysis results for Trait3**. Click here for file

## Declarations

This work and its publication were funded by EC-funded FP7 Project 3SR-245140. We also wish to acknowledge funding contributions from EADGENE_S, the BBSRC Institute Strategic Programme Grant at The Roslin Institute and the Scottish Government's Strategic Partnership for Animal Science Excellence (SPASE) initiative.

This article has been published as part of *BMC Proceedings *Volume 8 Supplement 5, 2014: Proceedings of the 16th European Workshop on QTL Mapping and Marker Assisted Selection (QTL-MAS). The full contents of the supplement are available online at http://www.biomedcentral.com/bmcproc/supplements/8/S5
