# Supplementary figures and images for: Regional Heritability Mapping to identify loci underlying genetic variation of complex traits
Source: BMC Proc. 2014 Oct 7;8(Suppl 5):S3. doi: 10.1186/1753-6561-8-S5-S3 (PMC4195407; doi:10.1186/1753-6561-8-S5-S3)

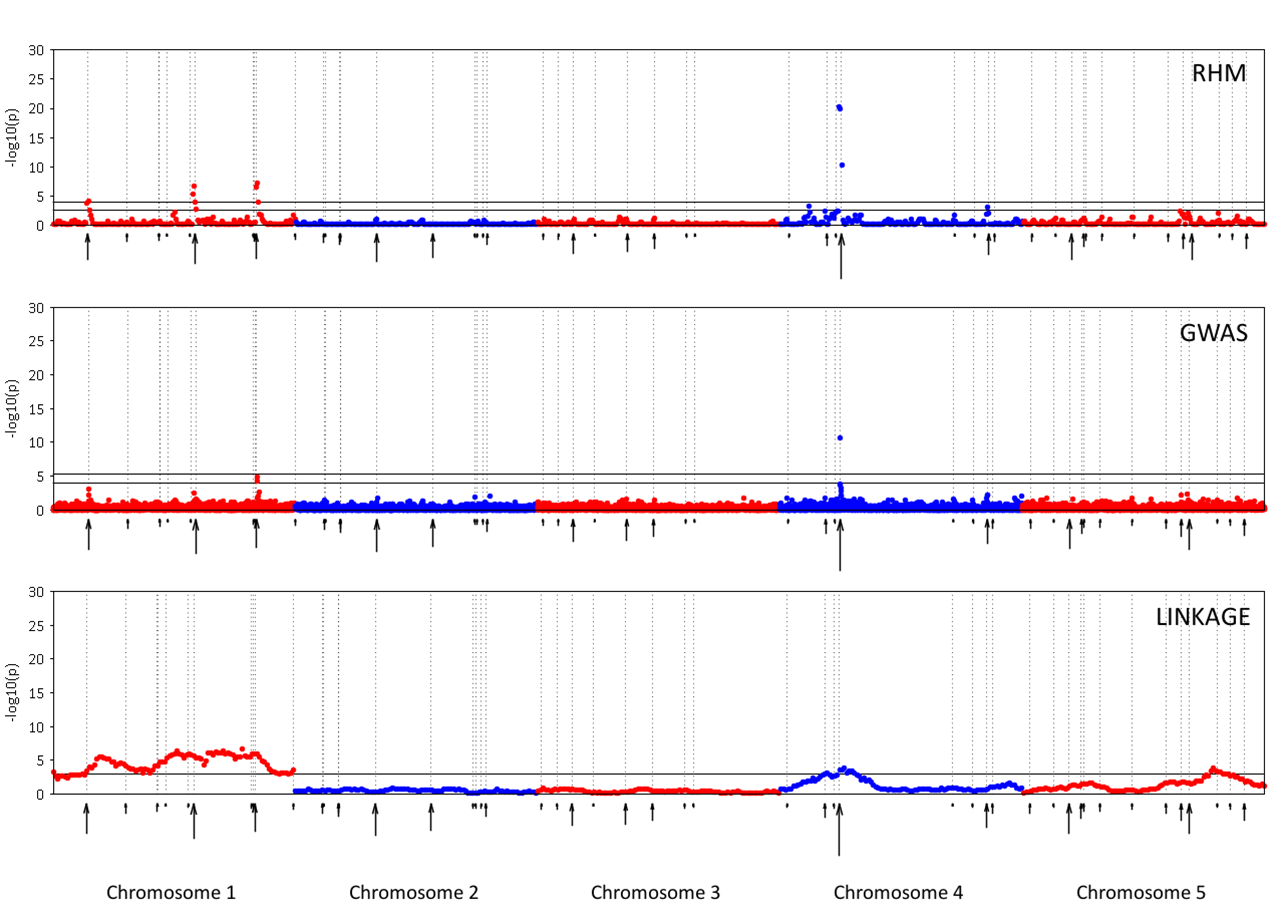

Supplement: Additional file 1 — Comparison among RHM, association and linkage analysis results for Trait1. [file 1753-6561-8-S5-S3-S1.png]

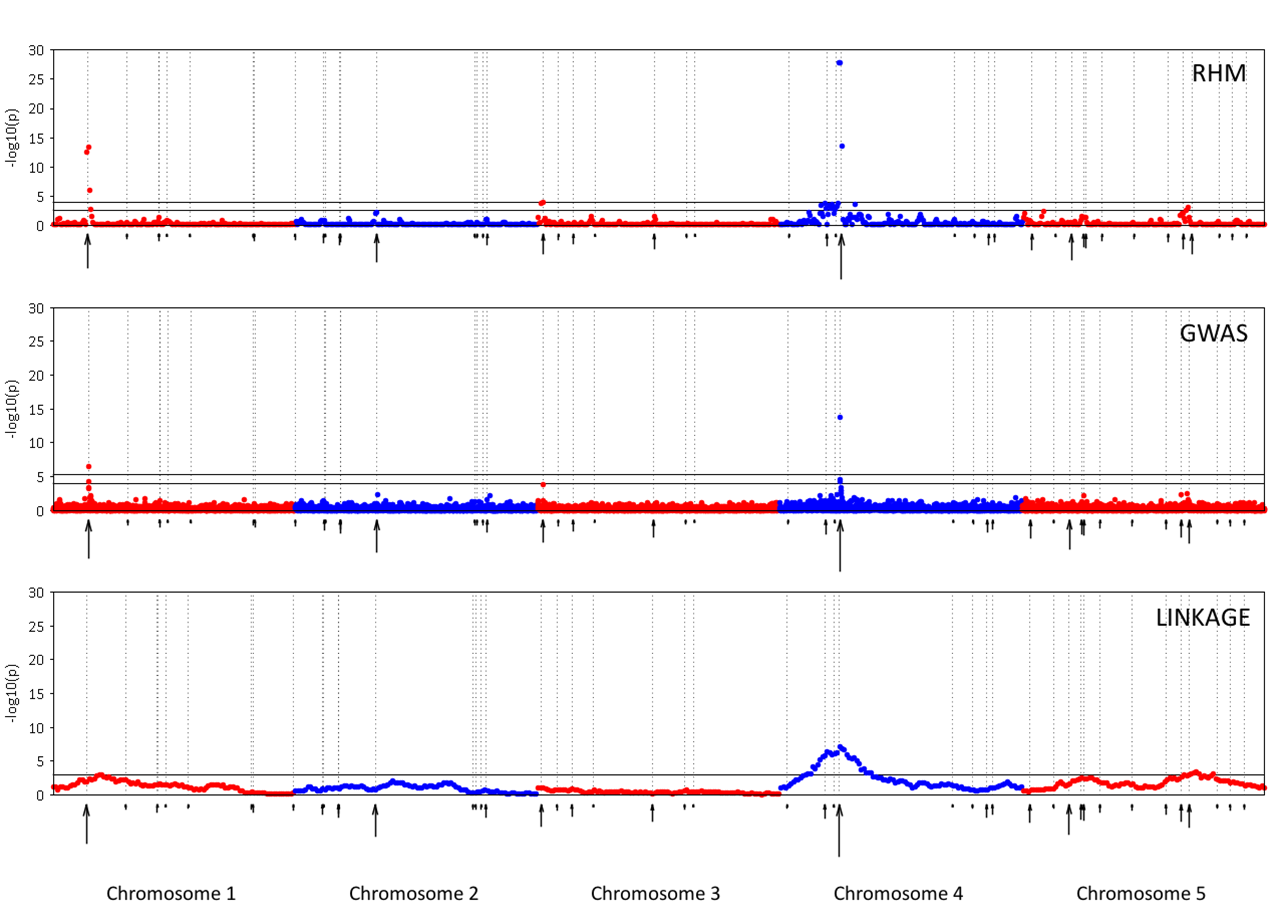

Supplement: Additional file 2 — Comparison among RHM, association and linkage analysis results for Trait2. [file 1753-6561-8-S5-S3-S2.png]

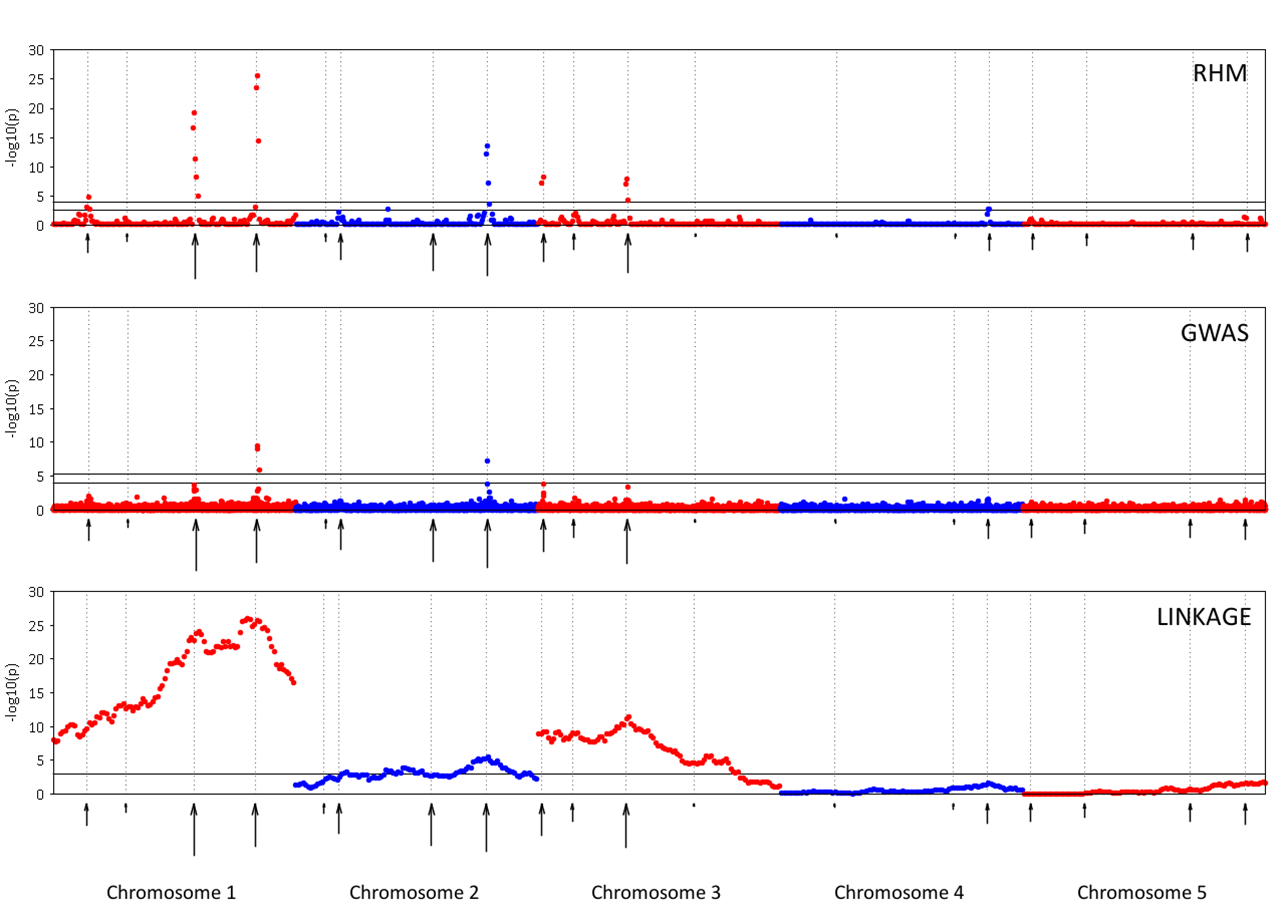

Supplement: Additional file 3 — Comparison among RHM, association and linkage analysis results for Trait3. [file 1753-6561-8-S5-S3-S3.png]
